# The Effect of Four Surface Treatment Methods on the Shear Bond Strength of Metallic Brackets to the Fluorosed Enamel

**Published:** 2015-09

**Authors:** Hooman Zarif Najafi, Vahid Moshkelgosha, Atefeh Khanchemehr, Akram Alizade, Ali Mokhtar

**Affiliations:** a Orthodontic Research Center, Dept. of Orthodontics, School of Dentistry, Shiraz University of Medical Sciences, Shiraz, Iran.; b Student Research Committee, School of Dentistry, Shiraz University of Medical Sciences, Shiraz, Iran.; c School of Dentistry, Tehran Islamic Azad University of Medical Sciences, Tehran, Iran.

**Keywords:** Air abrasion, Bond strength, Fluorosis, Laser

## Abstract

**Statement of the Problem:**

Some studies have reported the bond strength to be significantly lower in fluorotic enamels than the non-fluorosed.

**Purpose:**

The purpose of this study was to evaluate the shear bond streongth of metallic brackets to non-fluorosed and fluorosed teeth after different enamel conditioning.

**Materials and Method:**

A total of 176 freshly extracted human premolars (88 non-fluorosed and 88 fluorosed teeth) were used in this study for bonding the metallic brackets. Teeth with moderate fluorosis were used according to Thylstrup and Fejereskov index (TFI). Eighty non-fluorosed and 80 fluorosed teeth (TFI=4-6) were randomly divided into 8 equal groups of 20 teeth each. The remaining 16 teeth were used for scanning electron microscopy observation. The enamel surface was conditioned by 4 methods: acid etching  for 30 sec, acid etching for 120 sec, air abrasion followed by acid etching, and Er: YAG laser etching followed by acid etching. The morphology of etching patterns in different groups was studied under scanning electron microscope.

**Results:**

The shear bond strength of fluorosed teeth to the brackets was significantly lower than non-fluorosed ones (*p*= 0.003). The shear bond strength of laser-acid groups in both non-fluorosed and fluorosed teeth was significantly lower than other groups (*p*< 0.001). Weibull analysis indicated that the chance of failure under the applied force was different between fluorosed and non-fluorosed group. The scanning electron microscope observations revealed that the fluorosed teeth treated with phosphoric acid had fewer irregularities compared to non-fluorosed teeth. The most irregularities were detected in the teeth conditioned with phosphoric acid for 120 seconds.

**Conclusion:**

Fluorotic enamel adversely affects the bond strength of orthodontic brackets. None of the conditioning methods tested in this study could significantly improve shear bond strength of metallic brackets. Er: YAG laser conditioning followed by acid further reduced the bond strength in non-fluorosed and fluorosed teeth.

## Introduction

Fluoridation is considered among the most effective tools in prevention of dental caries.(1) However, excessive levels of fluoride in water supplies of different geographic areas have been responsible for clinically undesirable fluorosis.(2) Fluorosed enamel consists of an outer hypermineralized layer which is acid-resistant and an underlying hypomineralized porous layer.(3-4) Currently, direct bonding of brackets and attachments is one of the most commonly used techniques in fixed orthodontic treatment.(5) Meanwhile, obtaining a strong and reliable adhesive bond between the tooth enamel and orthodontic brackets is of great importance in orthodontic practice.(6)

While the routine method for conditioning the enamel surface is using phosphoric acid,(7) the hypermineralized enamel surface has been proved difficult to etch.(8) Some studies has found the bond strength to be significantly lower in fluorotic than the non-fluorosed enamels,(1, 9-10) while other studies declared no difference between the two.(5-6)

Several methods of enamel surface treatment have been recommended by different studies for reinforcement of the bond strength to the fluorosed enamel.(8, 11-12) One of these methods is grinding the enamel which results in enhanced surface roughness of the fluorosed tooth(13) and might reinforce the bond strength to enamel.(14) Similarly, extended enamel conditioning with phosphoric acid can remove the acid resistant hypermineralized surface layer.(15-16)

Air abrasion isanother method that has been applied in order to strengthen the bond strength to fluorosed teeth.(17) This method, in which the enamel surface is roughened, can be used during orthodontic treatments.(18-19) Some investigations(20-21) also showed that the lasers can be effective in enamel conditioning. Different types of laser such as Er:YAG (erbium-doped yttrium aluminum garnet), Nd:YAG (neodymium-doped yttrium aluminum garnet) and ErCr: YSGG have been used for enamel conditioning in orthodontics.(22) Er:YAG laser has been approved as an effective tool for hard tissue ablation.(23) Attrill *et al.*(20) supported the use of Er:YAG laser as an alternative to conventional acid etching. Although, Lee *et al.*(23) and Ferreira *et al.*(24)reported that the mean bond strength have not increased after laser etching followed by acid etching.

To the best of our knowledge, no studies have evaluated the effect of laser etching on the bond strength of fluorosed teeth. Concerning the previous controversial results regarding other enamel conditioning methods and high prevalence of dental fluorosis in some regions,(4) it seems necessary to scrutinize the best method of enamel surface treatment for bracket bonding in fluorosed teeth.

The aim of this study is to compare the shear bond strength (SBS) of metallic brackets to the fluorosed and non-fluorosed teeth after enamel preparation by use of four different surface conditioning methods: acid etching, prolonged etching, air abrasion combined with acid etching, and Er:YAG laser combined with acid etching. 

## Materials and Method

**Choosing and grouping the experimental teeth**

This *in vitro* study recruited 176 human premolars extracted for orthodontic reasons from the patients aged 20-40. These intact teeth had no caries, restoration, or chipping. Half of the samples (n=88) were non-fluorosed [Thylstrup and Fejereskov index (TFI) =0] and the other half were moderately fluorosed (TFI=4-6).(25) The fluorosed teeth were collected from endemic areas of fluorosis in southern parts of Iran.

All samples were cleaned by ultrasonic scaler (Dentsply International Inc.; York, USA) and were then polished for 20 seconds with non-fluoridated pumice and rubber cap. Finally, they were disinfected for about 24 hours through being plunged in distilled water comprising 0.1% thymol solution. After that, they were randomly divided into 8 equal groups of 20 teeth each. Groups 1 to 4 were non-fluorosed and groups 5 to 8 were fluorosed. The remaining 16 teeth were used for scanning electron microscopy (SEM) observation.

**Mounting and preparation of samples**

By using a mounting jig, the teeth were first embedded in a 2.6×2cm acrylic mold (Orthocryl; Dentaurum, Ispringen, Germany). The mounting jig was used to align the direction of debonding force parallel to the buccal surface of the teeth during the measurement of SBS. The buccal surfaces of the samples in groups 1 and 5 (acid-etch groups) were etched for 30 seconds with 37% phosphoric acid (Gel etch®; 3M Unitek, Monrovia, California, USA). After that, they were rinsed for 20 seconds with deionized water and were dried with oil-free air to leave a chalky white appearance. In groups 2 and 6 (prolonged-etch groups), the procedures were performed the same as what was done in groups 1 and 5, except that the teeth were etched for 120 seconds. The buccal surfaces of the teeth in groups 3 and 7 (air-abrasion and acid-etch groups) were sandblasted by use of 50-µm aluminum oxide particles for 5 seconds at 40-Ib pressure with micro-etcher (Micro–Etcher ERC II; Danville engineering, San Ramon, California, USA) held at 10-mm distance. It was followed by acid-etching with 37% phosphoric acid for 30 seconds before rinsing and drying the teeth. In order to determine the etching region in groups 4 and 8 (laser-acid etch groups), the buccal surfaces of the teeth were coated with nail varnish leaving a 4×4 mm window on the center of the crown as the laser irradiation area. Then the samples were irradiated with Er:YAG laser (DELight laser system; Continuum, Santa Clara, CA, USA) with a wavelength of 2.94 µm at 300 mj/pulse, 10pps, for 10 seconds. The laser beam application was directed manually from 1mm distance by using a 600µm optic fiber with water spray. After laser ablation, the teeth were etched with phosphoric acid for 30 seconds, then rinsed with deionized water for 20 seconds, and dried with oil-free air. 

**Bracket bonding**

After preparation, an adhesive primer (Transbond XT; 3m Unitek, Monrovia, Calif, USA) was applied to the etched surfaces of the samples in all groups. The adhesive paste was applied to the Ormco Mini 2000 (Ormco crop; Glendora, California, USA) premolar metal brackets (with mean surface area of 9.63mm(2)). The brackets were positioned on the teeth and seated with firm pressure to minimize the thickness of resin film. A probe was used to remove excess resin and then the teeth were light cured for 20 seconds by Ortholux LED (3M Unitek; Monrovia, California, USA).

**Measurement of the shear bond strength**

Before shear testing, all samples were kept in distilled water for 48 hours at room temperature. In order to determine the SBS, a mechanical testing machine (Instron Corp; Canton, Massachusetts, USA) applied an occluso-cervical force to the upper surface of the bracket between the bracket base and the upper wing. SBS was measured at a crosshead speed of 0.5 mm/ minute. The maximum force needed for bracket debonding was recorded in Newton (N) and the SBS was calculated through dividing the force value by the bracket base area (1 MPa = 1 N/mm(2)).

**Adhesive remnant index (ARI)**

After the experiment, an operator analyzed the brackets and teeth under a light stereomicroscope (Olympus SZ 6045 TR Zoomstere; Olympus Optical Co., Osaka, Japan) at 10X to determine the adhesive remnant index (ARI) according to Artun and Bergland.(26) The scoring criteria of the index were as (0) when no adhesive was remaining on the tooth surface, (1) when less than half the adhesive was remaining, (2) when more than half the adhesive was remaining, and (3) when the entire adhesive was remaining.

**Scanning electron microscopy (SEM) observation**

The roots were cut and then facial surfaces were treated similar to the enamel preparation that was performed for each of the 8 groups; 2 teeth for each enamel conditioning method. The prepared samples were washed with acetone solution for 10 minutes. Then, the enamel surfaces were sputter-coated with gold (SC-701AT; quick Auto coater, Sanyu Electron Inc, Tokyo, Japan) and observed under a SEM (JSM 5600 LV; JEOL, Tokyo, Japan) at 20KW and 1500X magnification.

**Statistical analysis**

The mean, standard deviation, minimum and maximum values were measured for all test groups. Two-way ANOVA was used to compare SBS among the groups. Weibull analysis was done to calculate the Weibull modulus, characteristic strength, and the required stress for 5% and 10% probabilities of bond failure. The Chi-square test was used to determine significant differences in the ARI scores among the groups. The statistical tests were performed with SPSS software, version 17.0 (SPSS Inc; Chicago, Illinois, USA), with the significance level set at 0.05. 

## Results

**Shear bond strength**

According to the results of two-way ANOVA test as displayed in Table 1, the SBS in the fluorosed teeth was significantly lower than non-fluorosed teeth (*p*< 0.05).

**Table 1:** Two-way ANOVA on the force (MPa) required for debonding the metal brackets from the enamel surface

**Source of variation**	**Sum of Squares**	Df*	**Mean Square**	***F*** ** ratio**	***P***
Fluorosis(or type of enamel)	117.056	1	117.056	9.371	.003
Methods	1579.698	3	526.566	42.155	.000
Fluorosis× Methods	17.248	3	5.749	.460	.710
Error	1898.670	152	12.491		
Corrected Total	3612.672	159			

*df: degree of freedom

Table 2 represents the SBS measurements including the mean, standard deviation, minimum and maximum values. The highest mean SBS was observed in prolonged etched groups (17.85±3.40MPa and 16.12±4.66MPa for non-fluorosed and fluorosed teeth, respectively). While, the lowest mean SBS was observed in the laser-acid etch groups for both non-fluorosed (9.06±1.83MPa) and fluorosed teeth (8.42±2.30 MPa). Post-hoc Tukey test revealed a significant difference between laser-acid etch groups and other groups in fluorosed and non-fluorosed teeth (*p*< 0.001). However, no significant difference was found among other groups (*p*> 0.05). Weibull data are also shown in Table 2. Generally, the non-fluorosed teeth had higher values of modulus when compared with fluorosed teeth. Characteristic bond strengths in this study ranged from 9.04 MPa in fluorosed teeth treated by laser followed by acid to 19.61MPa in non-fluorosed teeth treated by acid for 120 seconds, expecting that 63.21% of the brackets with this bond strength would fail.(27) The high values of correlation coefficient in the non-fluorosed prolonged etched group and fluorosed air abrasion-acid group (99%) demonstrated that the data strictly fitted the Weibull distribution function.

**Table 2:** The mean shear bond strength, standard deviation (SD), minimum and maximum values, and Weibull parameters for each group (n=20)

**Study groups**	**Mean ** **(MPa)**	**SD**	**Minimum**	**Maximum**		**Weibull analysis**				
						**Weibull modulus**	**Correlation coefficient**	**Characteristic strength (MPa)**	**Shear stress at 5% probability of failure (MPa)**	**Shear stress at 10% probability of failure (MPa)**
Non-Fluorosed teeth										
Acid etch	15.30	4.72	8.55	23.68		3.38	0.98	17.04	7.02	8.76
prolonged etch	17.85	3.40	11.47	22.12		5.28	0.99	19.61	11.18	12.81
Airabrasion+acid	16.87	3.10	11.73	21.18		5.53	0.98	17.60	10.29	11.72
Laser+acid	9.06*	1.83	6.70	12.25		5.18	0.95	9.88	5.57	6.35
fluorosed teeth										
Acid etch	13.19	3.04	8.65	18.28		4.48	0.98	14.53	7.49	8.79
prolonged etch	16.12	4.66	9.19	24.40		3.57	0.98	18.04	7.84	9.60
Airabrasion+acid	14.51	4.11	7.86	21.18		3.59	0.99	12.46	5.45	6.66
Laser+acid	8.42*	2.30	5.77		13.08	3.91	0.94	9.04	4.23	5.04

*shows statistically significant difference (*p*< 0.05) among the groups

**Scanning electron microscopy (SEM) observation**

**Figure 1:**
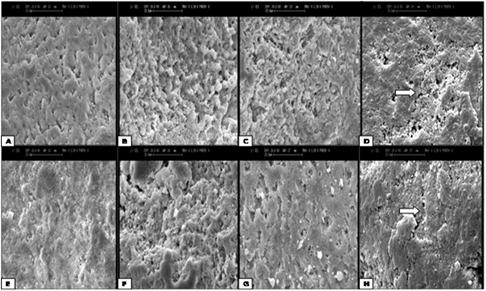
Scanning electron microscope images of non-fluorosed enamel surfaces (A, B, C, D) and fluorosed enamel surfaces (E, F, G, H): A and E: acid etch for 30 seconds, B and F: acid etch for 120 seconds, C and G: air abrasion followed by acid etch, D and H: laser ablated followed by acid etch. (Magnification ×1500) (The pointers show surface cracks created by laser).

SEM images of enamel surfaces treated with different methods in both non-fluorosed and fluorosed teeth are shown in figure 1. The fluorosed teeth, treated by phosphoric acid for 30 seconds showed fewer irregularities compared with non-fluorosed teeth (Figures 1a and 1e). Most irregularities were observed in the samples conditioned with phosphoric acid for 120 seconds (Figures 1b and 1f). Laser-acid etched teeth showed an indistinct etch pattern with surface cracking (Figures 1d and 1h).

**Adhesive remnant index (ARI)**

Modes of failure following the SBS test are summarized in Table 3. Chi-square test revealed a significant difference among the groups in terms of bond failure (*p*< 0.001). There were 90% and 100% frequency of ARI scores 0 and 1 in laser-acid etched groups in non-fluorosed and fluorosed teeth, respectively. But in other groups, the frequency of ARI scores was scattered.

**Table 3:** Distribution of adhesive remnant index (ARI) scorings in the study groups

**Study groups**	**ARI scores**				
	**0**	**1**	**2**	**3**	**Sum**
Non-Fluorosed teeth					
Acid etch	7(35%)	7(35%)	3(15%)	3(15%)	20(100%)
Prolonged etch	5(25%)	7(35%)	5(25%)	3(15%)	20(100%)
Airabrasion+acid	8(40%)	7(35%)	3(15%)	2(10%)	20(100%)
Laser+acid	15(75%)	3(15%)	2(10%)	0(0%)	20(100%)
fluorosed teeth					
Acid etch	8(40%)	7(35%)	3(15%)	2(10%)	20(100%)
Prolonged etch	5(25%)	3(15%)	3(15%)	9(45%)	20(100%)
Airabrasion+acid	3(15%)	7(35%)	3(15%)	7(35%)	20(100%)
Laser+acid	20(100%)	0(0%)	0(0%)	0(0%)	20(100%)

## Discussion

In this study the teeth were categorized according to the TFI.(25) This index shows relevance among the clinical form of fluorosis and the pathologic alterations in human teeth and is one of the best indices for assessment of severity of fluorosis.(28)The samples were obtained from patients aged 20-40 according to Ateyah and Akpata(8) who reported the bond strength to be significantly different between the patients under or above 40 years old.

The findings of this study demonstrated that SBS was significantly lower in fluorosed teeth compared with the non-fluorosed teeth (*p*=0.003). This may be related to the acid resistant superficial layer of the fluorosed teeth. In agreement with our results, some investigations(1, 9-10) indicated that there was a significant difference in the bond strength of fluorosed and non-fluorosed teeth. Although, Ng’ang’a *et al.*(5) and Isci *et al.*(6) found no differences in the bond strength of orthodontic brackets between fluorosed and non-fluorosed teeth etched with phosphoric acid. In our study, moderate fluorotic teeth extracted from the patients aged 20-40 years old were assessed; while the age range of patients and the fluorosis severity were different in the study conducted by Ng’ang’a *et al.*(5) and Isci *et al.*(6) Moreover, in contrast to the present study, Ng’ang’a *et al.*(5) used tensile force for measurement of the bond strength.

In our study, the highest mean SBS on debonding of both fluorosed and non-fluorosed teeth was found in prolonged etched groups, followed by air abrasion-acid etch groups. The laser-acid etch groups exhibited the lowest mean SBS on debonding (Table 2). In the prolonged etched groups, the etching time was raised up from 30 to 120 seconds to overcome the acid resistant layer and improving bond strength in the fluorosed teeth.(8) Opinya and Pameijer(16) evaluated the effect of prolonged etching of enamel on the bond strength of fluorosed teeth. They reported that extended enamel conditioning with phosphoric acid (120 seconds) could improve the bond strength in fluorosed teeth. Similarly, Ateyah and Akpata(8) reported that increasing the etching time to 120 seconds significantly increased the SBS of composite resin in mild and moderate fluorosed teeth of the patients aged less than 40 years; however, this was not the case in the teeth of older patients. But, findings of our study demonstrated that increasing the etching time from 30 to 120 seconds did not result in a significant increase in the SBS of orthodontic brackets for moderate fluorosed and non-fluorosed premolar teeth. Similar results were obtained by Silva Benitez *et al.*(15) who etched severely fluorosed molar teeth for 150 seconds. The different results yielded by different studies are probably due to the various severities of fluorosis and different etching time used in these studies.(8, 15) Ateyah and Akpata(8) used both anterior and posterior teeth (incisor, premolar, and molar) in their study and found that the fluoride content varied among different types of the teeth.(3) In addition, they grinded to flatten the hypermineralized surface layer before acid etching. The differences between the findings of Ateyah and Akpata(8) and our study may be related to the different techniques employed as mentioned above.

The findings of the current study showed that air abrasion followed by acid etching could increase SBS value compared with acid etching alone for both fluorosed and non-fluorosed teeth; although this difference was not statistically significant. Silva-Benitez *et al.*(15) detected that the use of air abrasion followed by acid in severe fluorosis provided adequate SBS for fixed orthodontic appliance, but the use of this treatment could not improve bond strength in case of moderate fluorosis. Suma *et al.*(19) stated that combining air abrasion with acid etching created greater SBS than acid etching alone in moderate to severe dental fluorosis regardless of the adhesion system used. They used air abrasion at air pressure of 80 PSI, and the teeth were etched for 60 seconds after air abrasion. The duration of acid etching and air pressure employed in this study was higher than our study and these may be the factors responsible for different result.

Findings of the current study demonstrated that bond strengths were significantly weaker when the tooth surfaces were prepared with the Er:YAG laser followed by acid etch compared with other enamel surface treatments; it was in line with the findings of Lee *et al.*(23) This might be due to the reduced surface area and pore volume in the enamel surface after laser ablation.(29) The lower bond strength observed in the laser followed by acid groups compared with acid alone in our study might be attributed to the acid resistant layer created in enamel surface after laser application as indicated in some investigations.(30-31) Some studies suggested that laser ablation could create modifications in chemical and crystalline structure. Reduction of carbonate amends the crystalline structure and subsequently causes resistance of enamel to acid dissolution.(30-31) This may be a factor for lower bond strength in laser-acid etch groups compared with acid etch groups. Although, Apel *et al.*(32) and Chimello *et al.*(33) found that enamel demineralization was not significantly different between Er:YAG laser and unlased teeth. Another explanation was presented by Ferreira *et al.*(24) who concluded that the residual thermal energy after Er:Yag laser-irradiation could change the structure of tooth surface by melting and packing the tooth components similar to that of glazing, and this change remained even after different acid etching times.(24) In contrast to our results, Dunn *et al.*(34) showed that laser followed by acid created better etch pattern and this pattern could be more effective for bonding. The histological investigation showed that different energies of Er:YAG laser could affect the quality of the resultant etched surface.(20)Therefore, the energy employed in different studies.(23-34) may be a causing factor for different results.

The Weibull analysis is a suitable means for predicting the likelihood of failure of bracket bonding.(35) The results of Weibull analysis in our study showed that the Weibull modulus of non-fluorosed teeth was generally higher than that of fluorosed teeth (Table 2). Higher Weibull modulus in the non-fluorosed teeth demonstrated high homogeneity of SBS values in these teeth.(36) Moreover, mean shear force at 5-10% probability of bond failure can be more clinically relevant than the mean strength values or high values.(37) Acceptable bond strength at 5% probability of failure is at least 5.4 MPa.(38) In the present study, the SBS of fluorosed teeth conditioned with laser followed by acid showed a lower shear stress level than 5.4 MPa at the 5% chance of failure. According to these findings it can be affirmed that laser followed by acid etching cannot be a good choice for enamel preparation.

Our SEM observations revealed that the non-fluorosed teeth treated for 30 seconds produced a pattern where the prism cores were eliminated and the adjacent zone did not conform to the prism structure (Figure 1a).However, this etching time for fluorosed teeth, that are more resistant to acid, created less roughness in the enamel surface (Figure 1e). The lower microporosities in the fluorosed enamel leads to weaker bond in these teeth. This claim is supported by some previous studies(16, 39) which showed that higher concentration of fluoride in teeth resulted in more resistance to acid etching and lower surface irregularity. Both the non-fluorosed and fluorosed enamels conditioned for 120 seconds showed very irregular surfaces with definite prominences and dents (Figures 1b and 1f). The non-fluorosed enamel treated with air abrasion and subsequent acid etching showed not only an irregular surface, but also a large number of holes probably created by the aluminum oxide (Figure 1c). The fluorosed enamel conditioned with air abrasion and subsequent acid etching displayed just the air abrasion effect (holes created by aluminum oxide) (Figure 1g). According to Olsen *et al.*,(40) this loss of enamel by aluminum oxide was irreversible, but with acid etching, organic component remained intact and it allowed the tooth surface to be remineralized. Laser-ablated enamels are seen in Figures 1d and 1h. These images indicate that laser cannot create distinct etch pattern in the enamel surface. Moreover, the residual thermal energy of Er:YAG laser leads to surface cracking and molten surface globules of enamel. Surface cracking created by laser can decrease the bond strength:(34, 41) and the bond strength data from the present study appeared to support this observation.

ARI evaluations showed that the de-bonded brackets in the laser-acid etch groups were separated from the resin-enamel interface. This type of bond failure was significantly different from the other types of bond failure occurred in other groups (*p*< 0.001). These results are similar to what was found by Martinez-Insua *et al.*(41)and Kameyama *et al.* regarding the fracture pattern.(42) We expected that this enamel preparation might not be able to give adequate surface wetting. In this fracture pattern, it is easy to remove the adhesive resin from the tooth surface; however, the probability of bond failure increases at low levels of applied stress.

## Conclusion

In conclusion, findings of the present study showed that enamel fluorosis significantly decreased the bond strength of orthodontic brackets. It was also found that none of the applied methods of prolonged etching, air abrasion combined with acid etching, and laser combined with acid etching could improve the SBS of metallic brackets in these teeth. Moreover, Er:YAG laser followed by acid etching significantly reduced the SBS when compared with the control acid etch group.

Future clinical investigations are recommended for enhancement of the bond strength in the moderate and also severely fluorosed teeth. Further studies are also required to be carried out on the use of other laser energy parameters for obtaining an acceptable prepared surface for bonding. 
